# Correspondence on “Effects of Temperature and DC Electric Fields on Perfluorooctanoic Acid Sorption Kinetics to Activated Carbon”

**DOI:** 10.1021/acs.est.4c07601

**Published:** 2024-10-14

**Authors:** Frank-Dieter Kopinke

**Affiliations:** Department of Technical Biogeochemistry, Helmholtz Centre for Environmental Research - UFZ, Leipzig D-04318, Germany

Jiao et al.^1^ have investigated the adsorption of perfluorooctanoic acid (PFOA) onto activated carbon (AC), specifically the influence of temperature and an external DC electric field. The authors derived key parameters of adsorption kinetics, such as intraparticle diffusivity *D*_eff_ and film mass-transfer coefficient *k*_fp_. However, in our opinion, the applied kinetic model is *a priori* inadequate, and consequently, the derived parameters are meaningless. In addition, we see significant inconsistencies in the presented experimental data. We substantiate this critical evaluation as follows.

The authors use eq 6 in their article^1^ to model the adsorption of PFOA on AC particles, which are arranged in a flow-through fixed bed. This equation is taken from ref 25 of their paper.^2^ However, in ref (2), the adsorption rate of particles in batch experiments is described, whereas in ref (1), this equation is applied for data from dynamic column experiments. There is a crucial difference between these two setups. In batch experiments, there is a time-dependent but uniform bulk-phase adsorbate concentration *C_t_* = *f*(*t*) ≠ *f*(*x*), whereas in a column setup, this concentration varies with time *t* (seconds) and displacement *x* (centimeters) along the column *C*_*t*,*x*_ = *f*(*t*, *x*). The use of *C*_outlet_/*C*_inlet_ in eqs 6 and 9 of ref (1) does not reflect the intracolumn concentration profile. Therefore, its application is incorrect, and consequently, all of the derived kinetic adsorption parameters are invalid. This also applies to the derived temperature and field effects. Thus, in strict terms, the further conclusions drawn in the article are unfortunately not worthy of consideration.

Nevertheless, it might be useful to inspect the original experimental data of the study. We checked the primary adsorption data presented in Figures 1, S2, and S3. These data are, in our view, inconsistent. In Figure 1 (and Figure S3E), we see that the PFOA loading of the AC in the fixed bed approaches a plateau (e.g., data series at 50 °C, no DC field, at ∼9 mg/g after 24 h, red filled symbols in Figure 1A). A plateau implies a complete breakthrough of PFOA, i.e., *C*_outlet_/*C*_inlet_ = 1, from ∼24 h on. From Figure S2A, we see that *C*_outlet_/*C*_inlet_ ≈ 0.55 rather than 1.0, at 24 h under identical conditions. These findings cannot be true simultaneously. The same inconsistencies hold for the majority of the experimental data presented in the addressed figures. Unfortunately, the authors do not present PFOA mass balances, which could possibly explain the inconsistencies of the data.

Looking at the next Figure, the reader is faced with the next problem. In panels A and B of Figure 2, the authors give a collection of kinetic parameters (*k*_fp_ and *D*_eff_), which allow Biot numbers *Bi* = *k*_fp_ × *R*/*D*_eff_, presented in Figure 2C, to be calculated. *Bi* quantifies the ratio between two rates: (i) the diffusion of adsorbate through the external stagnant solution layer and (ii) the intraparticle pore diffusion rate. *R* is the radius of the adsorbent particles (*R* = 0.275 mm). Upon performing this calculation, the reader is surprised that the self-calculated *Bi* values deviate by 2 orders of magnitude from those presented in Figure 2C by the authors. The reader can only speculate that the ordinate in Figure 2C is erroneously scaled.

The Biot number allows us to discriminate between the two sequential diffusion processes as possible rate-limiting steps of the adsorption. High values of *Bi* indicate that the external mass transfer is much faster than intrapore diffusion. In other words, the second of the two sequential steps is limiting for the total observable adsorption rate. In this case, the question of how one can determine meaningful film mass-transfer coefficients *k*_fp_ from such kinetic data arises. Ko et al.^2^ conducted a sensitivity analysis for the impact of *k*_fp_ on the position and shape of breakthrough curves in column experiments. They found that the calculated breakthrough curves do not have a significant response due to changes in *k*_fp_, even when this parameter varies by a factor of 2, with *Bi* = 264 as an example. In the presented column experiments, the Biot numbers were even higher, in fact much higher (∼10^4^). Nevertheless, Jiao et al.^1^ claim to deduce meaningful trends in *k*_fp_ values as *f*(*T*) and *f*(field strength). We have serious doubts regarding the validity of these conclusions.

It is striking that the two sets of kinetic parameters *k*_fp_ and *D*_eff_ both decrease with an increase in temperature (Figure 2). This “inverse” temperature dependence is untypical for diffusion-based processes, because the viscosity of the solvent, water, decreases with an increase in temperature, i.e., aqueous diffusion coefficients increase. *D*_eff_ is additionally affected by intrapore adsorption (retarded pore diffusion). For the exothermic adsorption of PFOA on AC, this retardation effect is attenuated with an increase in temperature (Table S3). However, the opposite tendency for *D*_eff_ = *f*(*T*) is shown in Figure 2B: it decreases with an increase in temperature. The unexpected temperature effects, independent of field effects, were not discussed by the authors. In our view, they are most likely due to the inappropriateness of the kinetic model used.

The authors also determined the adsorption isotherm parameters from batch experiments. Unfortunately, the original experimental data are not presented. The derived parameters (in the main text and in Table S3) are, in our view, erroneous and not compatible with the AC plateau loadings shown in Figure 1.

Finally, with regard to the investigated adsorbent material, Jiao et al.^1^ used a kind of activated carbon with an unusually small specific surface area of 28.4 m^2^/g and an exceptionally high particle density of 4.5 g/cm^3^ (cf. section S3). We do not know of any carbon modification with such a high density.

Summing up the practical outcomes of the study of Jiao et al.^1^ in Table 1, we find the maximum benefit of applying an external DC field on the PFOA adsorption on activated carbon amounts to 6–8% (at 10–20 °C, respectively) in terms of effective diffusion coefficient *D*_eff_. This is not a huge effect. Boosting the adsorption kinetics by enhancing the temperature of the water flow to be treated is usually not a favorable tool.
